# 1,4-Naphthoquinone Triggers Nematode Lethality by Inducing Oxidative Stress and Activating Insulin/IGF Signaling Pathway in *Caenorhabditis elegans*

**DOI:** 10.3390/molecules22050798

**Published:** 2017-05-13

**Authors:** Jia Wang, Guangzhi Zeng, Xiaobing Huang, Zhe Wang, Ninghua Tan

**Affiliations:** 1 State Key Laboratory of Phytochemistry and Plant Resources in West China, Kunming Institute of Botany, Chinese Academy of Sciences, Kunming 650201, China; wangjia@mail.kib.ac.cn (J.W.); g.zh_zeng@163.com (G.Z.); huangxiaobing@mail.kib.ac.cn (X.H.); wangzhe@cpu.edu.cn (Z.W.); 2 School of Traditional Chinese Pharmacy and State Key Laboratory of Natural Medicines, China Pharmaceutical University, Nanjing 211198, China; 3 YMU-HKBU Joint Laboratory of Traditional Natural Medicine, Yunnan Minzu University, Kunming 650500, China; 4 University of Chinese Academy of Sciences, Beijing 100049, China

**Keywords:** plant-parasitic nematode, phytochemical, nematocidal, 1,4-naphthoquinone, oxidative stress, insulin/IGF signaling pathway, *Caenorhabditis elegans*

## Abstract

Plant-parasitic nematodes are destructive pathogens causing enormous economic losses worldwide. With the withdrawal of fumigants, organophosphates and carbamates, pathogenic nematode control is more difficult. Phytochemicals are the plant secondary metabolites and are friendly for men and the environment. For developing new nematocidal candidates, we screened 790 phytochemicals using the model organism *Caenorhabditis elegans* and found 10 active compounds, 3 of which were further evaluated for their inhibitory activities against egg hatching of *C. elegans* and J2 *Meloidogyne incognita.* Among them, 1,4-naphthoquinone (1,4-NQ) was the only compound that could kill more than 50% of targets at 50 μg/mL, prompting us to investigate how 1,4-NQ triggers nematode lethality. In *C. elegans*, we observed that 1,4-NQ could influence reactive oxygen production, superoxide dismutase activity, and the heat-shock transcription factor (HSF)-1 pathway, which indicated that 1,4-NQ stimulated significant oxidative stress. Furthermore, using quantitative RT-PCR and transgenetic nematodes, we revealed that 1,4-NQ lethality was related to the Insulin/IGF signaling (IIS) pathway, and the effect of 1,4-NQ on IIS pathway related genes indicated that 1,4-NQ could activate this pathway and suppress the expression of DAF-16 target genes. The triggering of oxidative stress and activation of the IIS pathway indicated that 1,4-NQ operates through the generation of oxygen radicals, which can be lethal to *C. elegans*, thus making it an interesting lead compound for the development of future nematocides.

## 1. Introduction

Plant-parasitic nematode infections cause huge economic losses in global agriculture, which are estimated nearly at $157 billion annually [1,2]. Although, synthetic nematocides, such as fumigants, organophosphates, and carbamates, are effective for nematode control, their use is becoming increasingly restricted due to their side effects to the environment and human health, thus increasing the demand for more sustainable nematocides [3]. To develop reliable and effective nematocides, many studies focused on plant-parasitic nematode control, possessing lower influences on non-target organisms and biodegradability in a proper period of time [4]. Thus, plants and their naturally occurring byproducts provide alternatives to synthetic nematocides.

Plants can be an important resource for nematocidal compounds. For example, *Tagetes* spp., *Azadirachta indica,* and *Capsicum frutescens* have long been used for the control of plant-parasitic nematodes. Phytochemicals are the plant’s secondary metabolites and they play a key role in nematocidal plants, including essential oils, triterpenoids, alkaloids, glucosinolates, and phenolics [5]. Pyrethrins, derived from Asteraceae plants, not only exert a high toxicity for a wide range of pests and a low toxicity against mammals, but are also unstable in light, air, and moisture, making it easily degraded [5,6]. Azadirachtin, isolated from *A. indica*, has been used in commercial formulations that are effective against insects, mites, and nematodes [7,8,9]. Rotenone is the oldest pesticide in the world which is derived from Leguminosae and can be easily biodegraded in 5 to 7 h in field conditions [5]. All of these cases indicated that phytochemicals are friendlier to men and the environment than synthetic nematocides [10,11]. However, there are only a handful of plant-derived nematocides used in agriculture.

Parasitic nematodes must rely on their host to complete their lifecycle, making it difficult for the identification of nematocidal candidates in high throughput screening [12,13]. *Caenorhabditis elegans* is a free living nematode with a small size and a short lifespan, and offers a convenient and alternative model system for nematocidal compound screening and mechanism research [14,15]. Herein, using the nematode *C. elegans*, we found 10 active compounds by screening 790 distinct natural products and characterized the nematocidal mechanism of 1,4-naphthoquinone (1,4-NQ). Although, most quinones are redox cyclers that can cause a boost of reactive oxygen species (ROS) in cells [16,17,18], the underlying nematocidal mechanism of 1,4-NQ is lacking. Thus, we further performed assays on lethality, ROS intensity detection, superoxide dismutase (SOD) activity, heat-shock protein (HSF)-1, and Insulin/IGF signaling (IIS) pathway related gene expressions, mutant resistance, and DAF-16::GFP nuclear localization of *C. elegans* that exposed to 1,4-NQ, demonstrated that 1,4-NQ exerts nematode lethality by inducing oxidative stress and activating IIS pathway in *C. elegans*. This work not only discovers some plant nematocidal compounds, but also illustrates the nematocidal mechanism of 1,4-NQ, which will contribute to the future development of new nematocides.

## 2. Results and Discussion

### 2.1. Chemical Screening of Nematocidal Compounds

**Figure 1:**
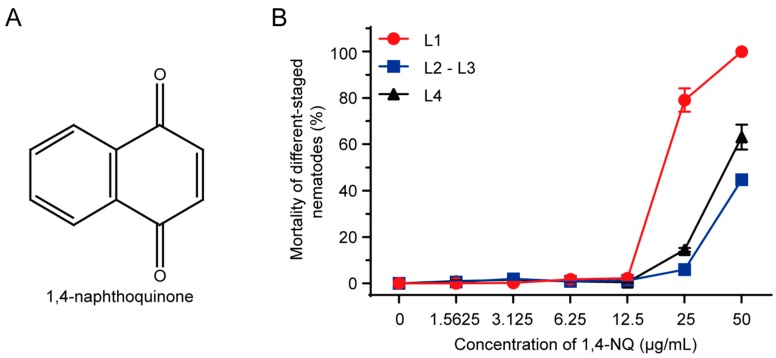
N2 L1s were the most sensitive to 1,4-naphthoquinone (1,4-NQ). (**A**) Chemical structure of 1,4-NQ; (**B**) The toxic effect of 1,4-NQ on different-staged wild type N2 nematodes. Different-staged larva were treated with various concentrations of 1,4-NQ for 48 h. Data presented as mean ± SEM and each experiment was repeated at least three times.

*C. elegans* is a useful and economical model for nematocidal compound screening [13]. Using the *C. elegans* as the model organism, we screened 790 phytochemicals against wild type N2 (N2) L4 worms at 50 μg/mL. After screening, rescreening, and evaluation assays, 10 active compounds were identified, three of which were further evaluated for affecting N2 egg hatching and *M. incognita* J2 toxicity. Among them, 1,4-NQ (Figure 1A) was found to be the only active compound that could not only kill N2 L4 nematodes (LC_50_ = 42.26 ± 2.53 μg/mL), and inhibit egg hatching of N2 (LC_50_ = 34.83 ± 0.58 μg/mL), but elicit toxicity on more than 50% of *M. incognita* at a concentration of less than 50 μg/mL (LC_50_ = 33.51 ± 0.21 μg/mL). For 1,4-NQ can be easily obtained from *Rubia wallichiana* and be degraded by the soil fungus *Phanerochaete chrysosporium* [19], we chose it for the following mechanism research, which will contribute to the future development of 1,4-NQ as a lead compound against plant-parasitic nematodes.

### 2.2. N2 L1 Was the Most Sensitive to 1,4-NQ

1,4-NQ is a kind of quinones found in plants, and its chemical structure was shown in Figure 1A. Dose-response curves were conducted for N2 L1, L2-L3, and L4 larva in acute toxicity assay (Figure 1B), and the LC_50_ values were 19.33 ± 0.60, 55.04 ± 0.11, and 42.26 ± 2.53 μg/mL, respectively, which drove us to choose N2 L1 as the research model to investigate the nematocidal mechanism of 1,4-NQ.

### 2.3. 1,4-NQ Stimulates ROS Production and Suppresses Elimination of ROS in C. elegans

**Figure 2:**
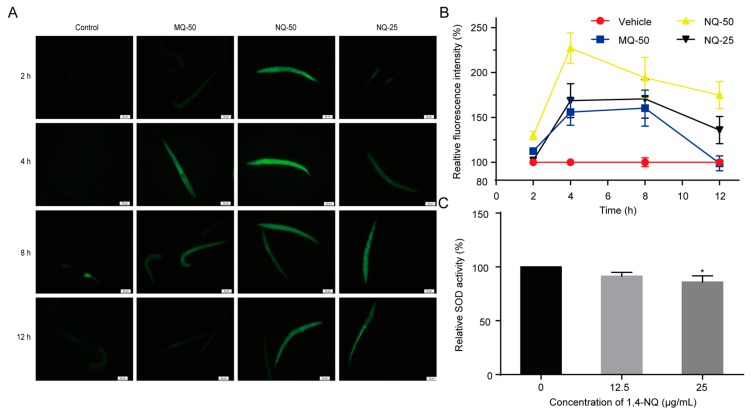
1,4-NQ stimulates reactive oxygen species (ROS) production and suppresses elimination of ROS in *C. elegans*. (**A**,**B**) Effects of 1,4-NQ on ROS production. Wild type N2 L1s were incubated with various concentrations of 1,4-NQ for 2, 4, 8, and 12 h, and then treated with 1μM carboxy-H_2_DCFDA for 30 min and photographed by fluorescence microscope, 2-methyl-1,4-naphthoquinone (menadione, MQ) as positive control; representative micrographs were shown in (**A**) and relative fluorescent intensities were shown in (**B**); MQ-50 means 50 μg/mL MQ, NQ-25 or NQ-50 mean 25 or 50 μg/mL 1,4-NQ, respectively. (**C**) 1,4-NQ suppresses SOD activity. Wild type N2 L1s were treated with 0, 12.5 and 25 μg/mL 1,4-NQ for 2 h. The worm lysates were prepared and subjected to analysis of SOD using assay kits. Data presented as mean ± SEM and each experiment was repeated at least three times. Significant differences between the control group and 1,4-NQ treated group, * *p* < 0.05 (one-way ANOVA followed by an LSD test).

Xenobiotic exposure may cause excess ROS production in cells and tissues, and induce intracellular redox homeostasis, such as irreversible oxidative modifications of lipid, protein, or DNA [20]. Previous studies revealed that quinones are redox cyclers and have the ability to stimulate ROS production in organisms. For example, 5-hydroxy-1,4-naphthoquinone (juglone) and 2-methyl-1,4-naphthoquinone (menadione, MQ) are used as ROS generators to induce oxidative stress in animals [21,22]. Thus, we used an indicator dye carboxy-H_2_DCFDA to measure the ROS levels of *C. elegans* exposed to various concentrations of 1,4-NQ or MQ, a positive control. Compared with the control, 1,4-NQ could excessively stimulate ROS generation in worms (Figure 2A,B). SOD is an anti-oxidant enzyme that converts superoxide to hydrogen peroxide and protects animals from ROS damage [23], so the influence of 1,4-NQ on SOD activity was investigated. The one-way ANOVA indicated that 1,4-NQ significantly altered SOD activity [*F*_(2,6)_ = 5.612, *p* < 0.05]. As shown in Figure 2C, SOD activity was significantly decreased after treating N2 L1 nematodes with 25 μg/mL 1,4-NQ. Taken together, these results indicated that 1,4-NQ not only stimulates ROS production but suppresses the elimination of ROS.

### 2.4. 1,4-NQ Activates HSF-1 Signaling Pathway in C. elegans

**Figure 3:**
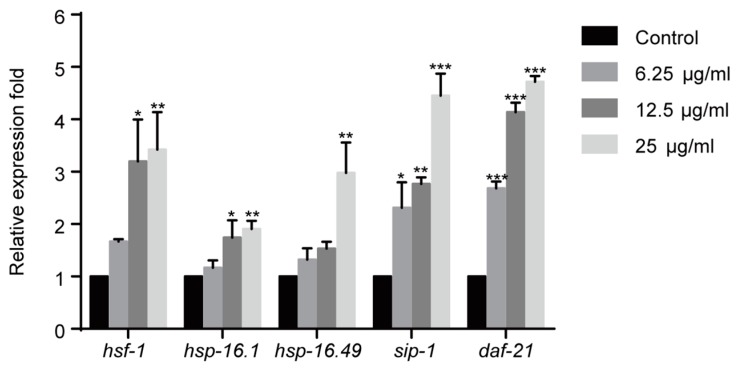
1,4-NQ activates HSF-1 pathway in *C. elegans*. Wild type N2 L1s were incubated with various concentrations of 1,4-NQ for 12 h, the expression of the HSF-1 pathway related genes, *hsf-1*, *hsp-16.1*, *hsp-16.49*, *sip-1,* and *daf-21* was measured by quantitative RT-PCR and normalized to *act-1* expression. Data presented as mean ± SEM and each experiment was repeated at least three times. Significant differences between the control group and 1,4-NQ treated group, *** *p* < 0.001, ** *p* < 0.01, * *p* < 0.05 (one-way ANOVA followed by an LSD test).

Heat shock proteins (HSPs) are chaperones that refold or recycle damaged proteins and inhibit protein aggregation [24,25]. As previously reported, redundant ROS could cause protein damage and aggregation, and activate heat-shock transcription factor (HSF)-1 pathway that regulates the expression of HSPs [20,24]. Therefore, the effect of 1,4-NQ on the expressions of HSF-1 pathway related genes was evaluated. Gene expressions were significantly altered among all experimental groups after 1,4-NQ treatment [*F*_(3,8)_ = 5.210, *p* < 0.05 for *hsf-1*; *F*_(3,8)_ = 5.772, *p* < 0.05 for *hsp-16.1*; *F*_(3,8)_ = 10.450, *p* < 0.01 for *hsp-16.49*; *F*_(3,8)_ = 19.619, *p* < 0.001 for *sip-1*; *F*_(3,8)_ = 180.515, *p* < 0.001 for *daf-21*]. The expression levels of *hsf-1*, *hsp-16.1*, *hsp-16.49*, *sip-1* and *daf-21* were all upregulated after exposure of 1,4-NQ, which suggested that 1,4-NQ activates HSF-1 pathway (Figure 3).

### 2.5. Effect of 1,4-NQ on IIS Pathway Related Gene Expressions in C. elegans

**Figure 4:**
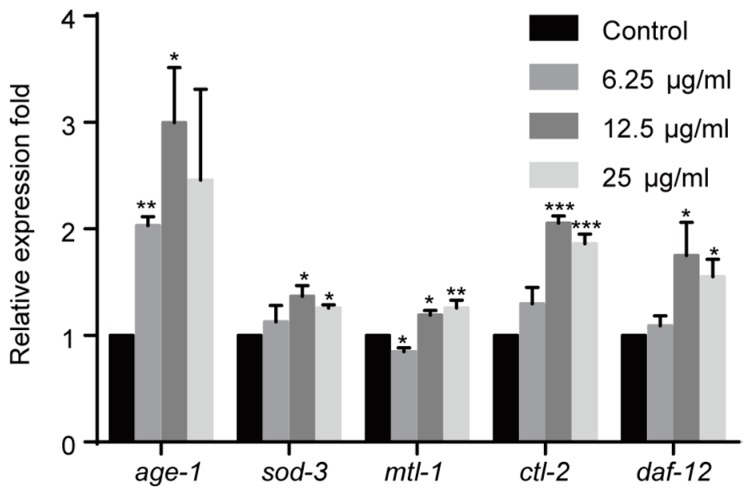
Effect of 1,4-NQ on the expression of IIS pathway related genes. L1 larva of wild type N2 were treated with various concentration of 1,4-NQ for 12 h at 20 °C, the expression of the IIS pathway related genes (*age-1*, *sod-3*, *mtl-1*, *ctl-2,* and *daf-12*) was measured by quantitative RT-PCR and normalized to *act-1* expression. Data presented as mean ± SEM and each experiment was repeated at least three times. Significant differences between the control group and 1,4-NQ treated group, *** *p* < 0.001, ** *p* < 0.01, * *p* < 0.05 (one-way ANOVA followed by an LSD or a Dunnett’s T3 test).

The IIS pathway plays a key role in innate immune system in *C. elegans.* Therefore we investigated the effect of 1,4-NQ on the IIS pathway. *age-1*, involved in the IIS pathway, could suppress the nuclear localization of DAF-16, which belongs to the forkhead transcription factors of FoxO family and regulates multiple stress-related gene expression [26,27]. We evaluated the effect of 1,4-NQ on the mRNA expression levels of IIS related genes: *age-1* and DAF-16 target genes (*sod-3*, *mtl-1*, *ctl-2*, and *daf-12*) [28,29]. One-way ANOVA analysis revealed that gene expressions were significantly altered among all experimental groups after 1,4-NQ treatment [*F*_(3,8)_ = 4.222, *p* < 0.05 for *age-1*; *F*_(3,8)_ = 4.407, *p* < 0.05 for *sod-3*; *F*_(3,8)_ = 16.627, *p* < 0.01 for *mtl-1*; *F*_(3,8)_ = 58.045, *p* < 0.001 for *ctl-2*; *F*_(3,8)_ = 4.477, *p* < 0.05 for *daf-12*]. The expression levels were all significantly increased in N2 L1 larva at 12.5 and 25 μg/mL 1,4-NQ, but were not significantly influenced at 6.25 μg/mL except that *age-1* was upregulated and *mtl-1* was downregulated (Figure 4). So it can be implied that 1,4-NQ influences the expression of the IIS pathway related genes in *C. elegans*.

### 2.6. Decreased Sensitivity of daf-2 Mutants and Increased Sensitivity of daf-16 Mutants to 1,4-NQ

**Figure 5:**
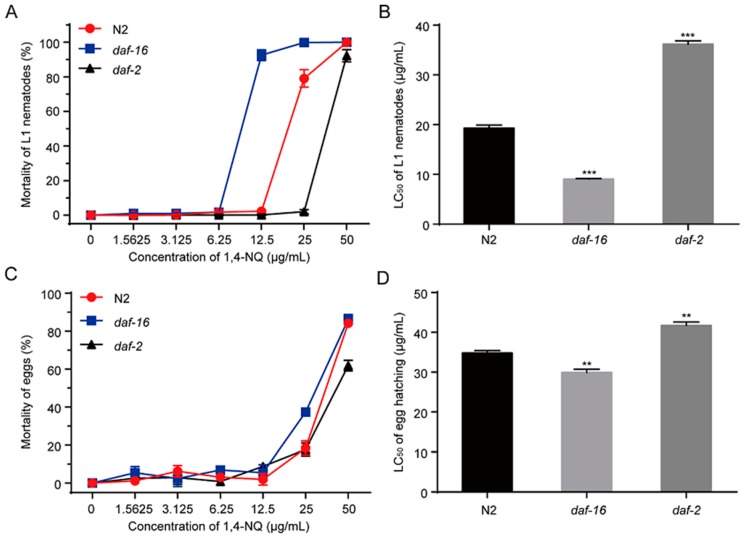
Mortality of 1,4-NQ on L1 larva and egg hatching of wild type N2, CF1038 *daf-16(mu86)I.* and CB1370 *daf-2(e1370)III.* L1 larva or embryos of wild type N2, CF1038 *daf-16(mu86)I.* and CB1370 *daf-2(e1370)III.* were incubated with various concentrations of 1,4-NQ for 48 h, and the mortality (A,C) and LC_50_ (B,D) were calculated by microscope. Data presented as mean ± SEM and each experiment was repeated at least three times. Significant differences between the 1,4-NQ treated N2 nematodes and the 1,4-NQ treated nematode mutants, *** *p* < 0.001, ** *p* < 0.01 (one-way ANOVA followed by an LSD test).

The IIS pathway in *C. elegans* is involved in metabolism, growth, development, behavior, longevity, reproduction, and stress resistance, in which DAF-2 and DAF-16 are the two key components [27]. In *C. elegans*, DAF-2 is the insulin/IGF-1 receptor ortholog and its activation can suppress nuclear localization of DAF-16 [30]. We tested the effects of 1,4-NQ on L1 lethality and egg hatching of N2, *daf-2* mutants (CB1370 *daf-2(e1370)III.*) and *daf-16* mutants (CF1038 *daf-16(mu86)I.*). Compared to N2 nematodes, *daf-2* mutants are more resistant to 1,4-NQ but *daf-16* mutants are more sensitive [*F*_(2,6)_ = 702.137, *p* < 0.001 for L1 lethality; *F*_(2,6)_ = 60.928, *p* < 0.001 for egg hatching] (Figure 5). The LC_50_ values of L1 lethality and egg hatching of *daf-2* mutants were significantly increased (LC_50_ = 36.20 ± 0.66 μg/mL and LC_50_ = 41.71 ± 0.88 μg/mL, respectively) compared to N2 (LC_50_ = 19.33 ± 0.60 μg/mL and LC_50_ = 34.83 ± 0.58 μg/mL, respectively), while *daf-16* mutants were decreased (LC_50_ = 9.07 ± 0.11 μg/mL and LC_50_ = 29.95 ± 0.77 μg/mL, respectively) (Figure 5B,D). These results suggested that DAF-16 is related to nematodes resistance against 1,4-NQ.

### 2.7. 1,4-NQ Stimulates DAF-16::GFP Localization in C. elegans

**Figure 6:**
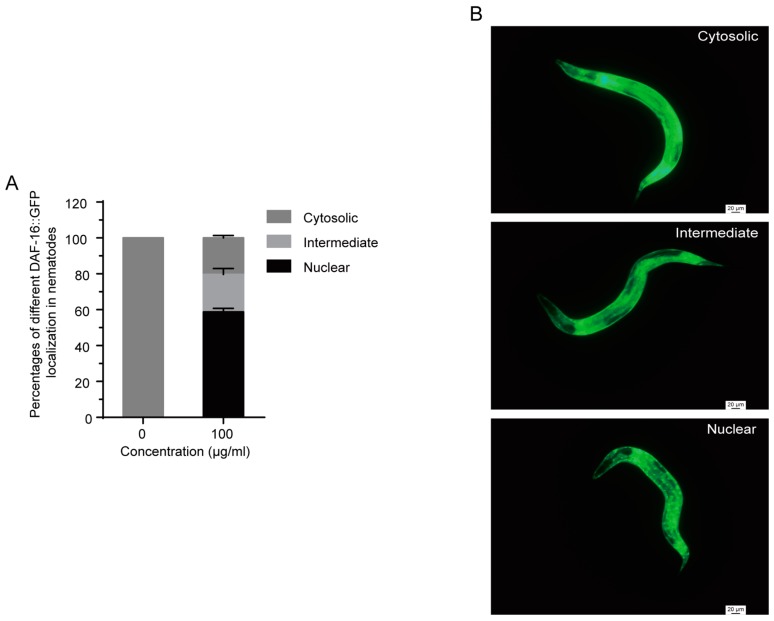
Effect of 1,4-NQ on DAF-16::GFP nuclear localization. L4 larva of transgenic strain TJ356 *daf-16(zls356)IV*. were placed on NGM plates with or without 100 μg/mL 1,4-NQ at 20 °C for 4 h, and then were photographed and monitored with fluorescence microscope (**A**); representative micrographs of DAF-16::GFP localization in cytosolic, intermediate and nuclear (**B**). Data presented as mean ± SEM and each experiment was repeated at least three times.

DAF-16 translocates from the cytoplasm to the nucleus when it was activated [31], so we performed a DAF-16::GFP localization assay to investigate whether 1,4-NQ induced the nuclear translocation of DAF-16 using transgenetic nematodes TJ356 *daf-16(zls356)IV*. As shown in Figure 6, a significant DAF-16 localization in nuclear was observed by treatment with 100 μg/mL 1,4-NQ for 4 h.

### 2.8. Discussion

Plants have been widely used in plant-parasitic control, but only a handful of plant-derived nematocides are used in the world. In order to develop new plant nematocidal candidates, we took advantage of the natural compound library in our lab by screening 790 phytochemicals using *C. elegans* or. J2 *M. incognita*, which led to 1,4-NQ, isolated from the roots and rhizomes of *Rubia wallichiana*, as the candidate for nematocidal mechanism research.

Most quinones are redox cyclers and could stimulate oxidative stress in organisms. In our study, 1,4-NQ could excessively stimulate ROS production in worms and suppress SOD activity (Figure 2). Therefore, the outburst of oxidative stress induced by 1,4-NQ in worms may be a comprehensive consequence of ROS production and the disruption of the ROS defense system. Previous studies revealed that high level of oxidative stress could trigger cell death by inducing oxidative damage to protein, lipids, and DNA [20]. And we speculated that excessive ROS and enginery dysfunction in ROS elimination might be the main reason for the death of nematodes induced by 1,4-NQ. HSPs can bind to damaged or unfolded proteins and mediate aggregation proteins to be refolded or recycled [24,25]. And they are regulated by the HSF-1 signaling pathway which could be activated by protein damage [24]. Thus, we tested the expression levels of several HSP genes (*hsf-1*, *hsp-16.1*, *hsp-16.49*, *sip-1,* and *daf-21*) and all of the tested genes were upregulated after 1,4-NQ treatment (Figure 3). The requirement of HSF-1 and HSPs suggests protein damage in nematodes. Therefore, we postulate that protein damage occurs during the exposure to 1,4-NQ, which is a significant factor contributing to the death of *C. elegans*.

The IIS pathway, a key component of an innate immune system, is involved in stress responses, so we investigated whether 1,4-NQ influences the expression of IIS pathway related genes. We found that the mRNA levels of DAF-16 targeted genes *sod-3*, *mtl-1*, *ctl-2,* and *daf-12* were significantly increased in N2 treated with 1,4-NQ at 12.5 and 25 μg/mL, which suggested the nuclear localization of DAF-16. However, we also discovered that *age-1* was also upregulated, furthermore, after treatment with 6.25 μg/mL 1,4-NQ, *age-1* gene was still upregulated and *mtl-1* gene was down regulated, with other genes not significantly influenced (Figure 4). Mutants in *age-1* are more resistant to ROS generator paraquat, and overexpression of *age-1* might suppress nuclear localization of DAF-16 and the expression of DAF-16 target genes [26,32]. So these data suggested that the IIS pathway might be activated and the expression of DAF-16 target genes was suppressed.

To investigate whether IIS was activated by 1,4-NQ treatment, experiments were carried out to test if DAF-16 is required for 1,4-NQ-induced response. We found that compared with N2 L1s, *daf-2* mutants are more resistance to 1,4-NQ, but *daf-16* mutants are more sensitive (Figure 5). We also observed a significant DAF-16 localization in nuclear after treatment of 100 μg/mL 1,4-NQ, which implied that 1,4-NQ could induce DAF-16 nuclear localization (Figure 6). All of these indicated that DAF-16 is required for nematode resistance against 1,4-NQ.

Previous studies showed that DAF-16/FoxO is not only regulated by the IIS pathway, but it also can be activated by other activators, such as oxidative stress and HSF-1 [28,33]. In other words, damage not only occurs through the direct detrimental effects of xenobiotics, but also through the side effect elicited by the defense responses toward xenobiotic. Thus, we could speculate that DAF-16 localization was modulated by oxidative stress and the HSF-1 signaling pathway that was induced by high concentration of 1,4-NQ treatment (Figure 2), which resulted in resistance of *daf-2* mutants against 1,4-NQ and nuclear localization of DAF-16. Thus, the suppressive activity of *age-1* on DAF-16 nuclear localization was overlapped by other DAF-16 activators, which resulted in the upregulation of *age-1* and DAF-16 target genes at 12.5 and 25 μg/mL. However, at a low concentration, modulation of other activators on DAF-16 nuclear localization is not obvious and the effect of 1,4-NQ on the IIS pathway’s activating is emerging, which results in the upregulation of *age-1* and down regulation of the DAF-16 target gene *mtl-1*. Taken together, 1,4-NQ probably activated the IIS pathway and resulted in suppression of DAF-16 nuclear localization, thereby contributing to nematode death.

**Figure 7:**
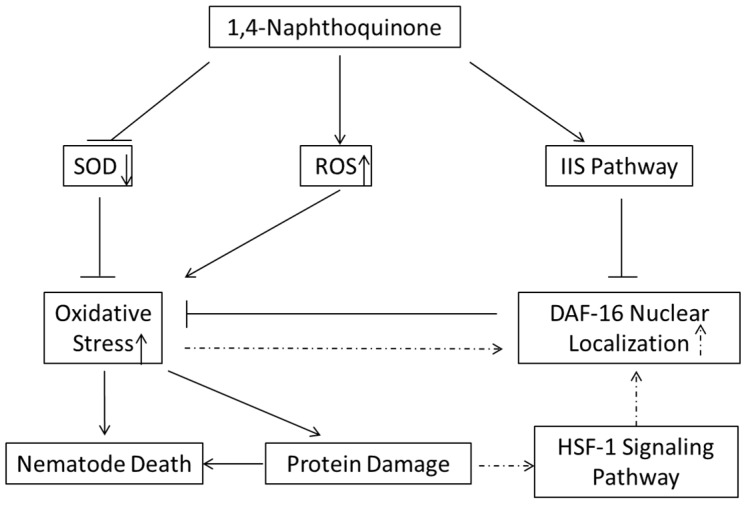
Schematic diagram of the proposed nematocidal mechanism of 1,4-NQ. Solid line, virulence mechanisms of 1,4-NQ; dashed line, resistance response of nematodes.

In conclusion, we screened 790 phytochemicals using *C. elegans* or J2 *M. incognita* and revealed the nematocidal mechanism of candidate 1,4-NQ (Figure 7). In this work, we found that 1,4-NQ exerts nematocidal activity by inducing oxidative stress and activating the IIS pathway to suppress resistant gene expression, which may be developed into a more environmentally friendly nematocides.

## 3. Materials and Methods

### 3.1. Chemical Compounds

All of these chemicals were obtained from the library of phytochemicals in our lab. 1,4-Naphthoquinone was isolated from the roots and rhizomes of *Rubia wallichiana*. Menadione was purchased from Aladdin Industrial Cooperation, Shanghai, China.

### 3.2. Nematode Strains and Culture Methods

Strains N2 (Bristol, wild type), CB1370 *daf-2(e1370)III*., CF1038 *daf-16(mu86)I*. and TJ356 *daf-16(zls356)IV*. were purchased from Caenorhabditis Genetics Center (CGC). All strains were maintained at 20 °C and grew on nematode growth medium (NGM) plates seeded with *Escherichia coli* OP50 [34]. Embryos were collected after gravid nematodes were treated with hypochlorite bleaching. After hatching for 12 h, L1 nematodes were moved to NGM plates with *Escherichia coli* OP50, L2-L3 larva were then collected after being cultured for about 24 h and L4 worms were collected after being cultured for about 48 h [35].

Infected soil was obtained from Yuxi, Yunnan Province, China and used to establish a population of *M. incognita* on tomato. Egg sacks were picked from heavily infected roots using a needle. These egg sacks were sterilized by 1% NaClO, and then washed three times in sterilized water. The sterilized egg sacks were placed on a 9 cm diameter sterile Petri dish and incubated at 28 °C in sterilized water. After hatching for 48 h, second juveniles J2 were removed and collected for liquid-based chemical screening.

### 3.3. Chemical Screening

Screening and rescreening: wild type N2 L4 worms were collected in M9 buffer and arrayed at 70 nematodes per well in 96-well plates, 100 μL per well. Chemicals were added to each well at a final concentration of 50 μg/mL (0.5% *v*/*v* DMSO), with DMSO as controls. Evaluation screening: wild type N2 L4 or J2 *M. incognita* were collected in M9 buffer or water and arrayed at 300 nematodes per well in 24-well plates, 400 μL per well. Chemicals were added to each well at a final concentration of 0, 3.125, 6.25, 12.5, 25 and 50 μg/mL (0.5% *v*/*v* DMSO), with DMSO as controls. Egg hatching assay: wild type N2 embryos were collected immediately after bleaching and dispensed into 24-well plates with 300 eggs, 400 μL per well, with a final concentration of 0, 3.125, 6.25, 12.5, 25 and 50 μg/mL (0.5% *v*/*v* DMSO), with DMSO as controls. All the results were conducted after 48 h exposure of phytochemicals.

### 3.4. ROS Measurement

Synchronized wild type N2 L1 nematodes were treated 0, 25, 50 μg/mL 1,4-NQ or 50 μg/mL MQ (positive control) for 2, 4, 8, and 12 h, respectively, and then washed three times in M9 buffer. The washed test worms were transferred to 1 mL of M9 buffer containing 1μM carboxy-H_2_DCFDA. After incubation for 30 min, nematodes were washed three times in M9 buffer, and then mounted on a glass slide for examination with a laser scanning confocal microscope (Leica, Leica DM5500B, Bensheim, Germany) at 488 nm of excitation wavelength and 510 nm of emission filter. The fluorescent intensities were semi-quantified as relative fluorescent units (RFU). More than 50 worms were examined per replicate.

### 3.5. Measurement of SOD Activity

About 50,000 synchronized wild type N2 L1 nematodes were treated with 0, 12.5, and 25 μg/mL 1,4-NQ, respectively and cultured at 20 °C for 2 h. Then, the worms were washed three times in M9 buffer. The worms were collected and lysed for analysis of SOD. SOD activity was measured at 450 nm following the manufacturer’s protocols of commercially available kits (Nanjing Jiancheng Bioengineering Institute, Nanjing, China).

### 3.6. Reverse Transcription-Polymerase Chain Reaction (RT--PCR) Analyses

About 100,000 synchronized wild type N2 L1 nematodes were treated with 0, 6.25, 12.5, and 25 μg/mL 1,4-NQ and cultured at 20 °C. After treatment for 12 h, worms were washed three times in M9 buffer and collected for RNA isolation. Total RNA was isolated using Trizol reagent (Invitrogen Corp., Carlsbad, CA, USA) and converted to cDNA using PrimeScript™ RT reagent Kit (TaKaRa, Tokyo, Japan). The RT-PCR was performed using iTaq™ Universal SYBR^®^ Green Supermix (Bio-rad) and Bio-Rad CFX Manager 3.1. Primer sequences are listed in Table S1.

### 3.7. Mutant Resistance Assay

About 300 L1s or embryos of wild type N2, CB1370 *daf-2(e1370)III*. and CF1038 *daf-16(mu86)I*. were collected in M9 buffer and added to 24-well plates, 400 μL per well. 1,4-NQ was added to each well at a final concentration of 0, 3.125, 6.25, 12.5, 25, and 50 μg/mL (0.5% *v*/*v* DMSO, DMSO as controls). The results were conducted after treatment with 1,4-NQ for 48 h.

### 3.8. DAF-16::GFP Nuclear Localization

Synchronized L4 larva of transgenic strain TJ356 *daf-16(zls356)IV*. were used for the observation of DAF-16::GFP localization. Worms were placed on NGM plates with or without 100 μg/mL 1,4-NQ at 20 °C for 4 h and then monitored with a fluorescence microscope (Leica, Leica DM5500B, Bensheim, Germany). The accumulation of DAF-16::GFP localization was scored as described previously [36]. More than 30 worms were conducted per replicate.

### 3.9. Statistical Analysis

All experiment data was repeated at least three times and expressed as mean ± SEM. Data was analyzed using SPSS 16.0 (SPSS Inc., Chicago, IL, USA). Statistical significance was estimated with a one-way ANOVA followed by an LSD (equal variances assumed) or a Dunnett’s T3 (equal variances not assumed) *post-hoc* test to determine significant differences between groups. *p* < 0.05 was considered as significant value.

## Supplementary Materials

The following are available online at www.mdpi.com/1420-3049/22/5/798/s1.
