# Supplementary material for: 1,4-Naphthoquinone Triggers Nematode Lethality by Inducing Oxidative Stress and Activating Insulin/IGF Signaling Pathway in Caenorhabditis elegans
Source: Molecules. 2017 May 13;22(5):798. doi: 10.3390/molecules22050798 (PMC6154497; doi:10.3390/molecules22050798)
Supplement: Supplementary file 1 [file molecules-22-00798-s001.pdf]

Table S1. Primer sequence

**Gene name**	**Primer sequence**
act-1	f: 5′-GTGTGACGACGAGGTTGCCGCTCTTGTTGTAGAC-3′
	r: 5′-GGTAAGGATCTTCATGAGGTAATCAGTAAGATCAC-3′
age-1 (B0334.8)	f: 5'-TCCTTGTTCACCTTGATGCTCGGA-3'
	r: 5'-TTGACTGCGTGGAAGAGCCAATTC-3'
sod-3	f: 5'-GCTGCAATCTACTGCTCGCACTGCTTCAAAGC-3′
	r: 5′-GGCAAATCTCTCGCTGATATTCTTCCAGTTGGC-3′
mtl-1	f: 5′-ATGGCTTGCAAGTGTGACTGCAAAAACAAGC-3′
	r: 5′-TTAATGAGCCGCAGCAGTTCCCTGGTGTTGATGGG-3′
ctl-2 (Y54G11A.5)	f: 5'-ACTTCGCTGAGGTTGAACAATCCG-3'
	r: 5'-TTTGGTCCAAGGCGGTGGAAATGA-3'
daf-12 (F11A1.13a)	f: 5'-TCCAATGCCAGCTGAAACAACACC-3'
	r: 5'-TGGAATGGCTGACACGGTTGAATG-3'
hsf-1	f: 5′-GCGGCTCCGTATAAGAATGCGACTAGGC-3′
	r: 5′-TTAAACCAAATTAGGATCCGATGGACTTGGAGTAC-3′
hsp-16.1	f: 5′-GTCACTTTACCACTATTTCCGTCCAGCTCAACGTTC-3′
	r: 5′-CAACGGGCGCTTGCTGAATTGGAATAGATCTTCC-3′
hsp-16.49	f: 5′-GCTCATGCTCCGTTCTCCATATTCTGATTCAAATGC-3′
	r: 5′-GCAACAAAATTGATCGGAATAGAACGTGATGAG-3′
sip-1	f: 5′-ATGTCTTCTCTCTGCCCATACACTGGCCG-3′
	r: 5′-TTAGTGCTTTCCGGTGGTGGTGGTGCTGG-3′
daf-21 (C47E8.5)	f: 5'-ACCTTGGAACCATTGCCAAGTCTG-3'
	r: 5'-ATCGGCGACAAGGAAAGCAGAGTA-3'
